# Program death ligand‐1 immunocytochemistry in lung cancer cytological samples: A systematic review

**DOI:** 10.1002/dc.24955

**Published:** 2022-03-16

**Authors:** Swati Satturwar, Ilaria Girolami, Enrico Munari, Francesco Ciompi, Albino Eccher, Liron Pantanowitz

**Affiliations:** ^1^ Department of Pathology The Ohio State University Columbus Ohio USA; ^2^ Division of Pathology Bolzano Central Hospital Bolzano Italy; ^3^ Pathology Unit, Department of Molecular and Translational Medicine University of Brescia Brescia Italy; ^4^ Computational Pathology Group, Department of Pathology Radboud University Medical Center Nijmegen Netherlands; ^5^ Department of Pathology and Diagnostics University and Hospital Trust of Verona Verona Italy; ^6^ Department of Pathology University of Michigan Ann Arbor Michigan USA

**Keywords:** cancer, concordance, cytology, FNA, immunohistochemistry, lung, PD1/PDL1

## Abstract

In this era of personalized medicine, targeted immunotherapies like immune checkpoint inhibitors (ICI) blocking the programmed death‐1 (PD‐1)/program death ligand‐1 (PD‐L1) axis have become an integral part of treating advanced stage non‐small cell lung carcinoma (NSCLC) and many other cancer types. Multiple monoclonal antibodies are available commercially to detect PD‐L1 expression in tumor cells by immunohistochemistry (IHC). As most clinical trials initially required tumor biopsy for PD‐L1 detection by IHC, many of the currently available PD‐1/PD‐L1 assays have been developed and validated on formalin fixed tissue specimens. The majority (>50%) of lung cancer cases do not have a surgical biopsy or resection specimen available for ancillary testing and instead must rely primarily on fine needle aspiration biopsy specimens for diagnosis, staging and ancillary tests. Review of the literature shows multiple studies exploring the feasibility of PD‐L1 IHC on cytological samples. In addition, there are studies addressing various aspects of IHC validation on cytology preparations including pre‐analytical (e.g., different fixatives), analytical (e.g., antibody clone, staining platforms, inter and intra‐observer agreement, cytology‐histology concordance) and post‐analytical (e.g., clinical outcome) issues. Although promising results in this field have emerged utilizing cytology samples, many important questions still need to be addressed. This review summarizes the literature of PD‐L1 IHC in lung cytology specimens and provides practical tips for optimizing analysis.

## 1 INTRODUCTION

Non‐small cell lung carcinoma (NSCLC) accounts for 80%–85% of cases and primarily comprises adenocarcinoma, squamous cell carcinoma and a not otherwise specified (NOS) category.^1^ The overall 5‐year survival rate for NSCLC is only 17%. Current treatment options include surgical resection, neoadjuvant chemotherapy and targeted therapies.^1^, ^2^, ^3^ Along with molecular prognostic biomarkers, programmed death‐1 (PD‐1)/program death ligand‐1 (PD‐L1) immunohistochemistry (IHC) has become an integral part of standard treatment regimens for NSCLC. T‐lymphocytes express PD‐1 and tumor cells express either PD‐L1 or PD‐L2. Binding of PD‐1 to PD‐L1 leads to increased apoptosis of activated tumor reactive T‐cells that, in turn, promotes growth of tumor cells by an immune escape mechanism.^4^, ^5^ Novel targeted immunotherapies block this pathway, thereby leading to tumor cell death. The use of such immune checkpoint inhibitors (ICIs) for NSCLC has emerged as one of the most promising cancer treatments.

PD‐L1 expression can be detected by IHC on tissue specimens or immunocytochemistry (ICC) on cytology samples, as well as by flow cytometry, fluorescent in‐situ hybridization (FISH) or molecular testing. Of these tests, IHC or ICC remain the most cost‐effective and most commonly used tests for the detection of PD‐L1 expression. Studies have shown better prognosis with anti‐PD‐1/PD‐L1 therapies for certain cancers compared to platinum‐based chemotherapies.^6^ As most clinical trials initially required tumor biopsy for PD‐L1 detection by IHC, most of the currently available PD‐1/PD‐L1 assays have been developed and validated on formalin fixed tissue and not alcohol fixed cytology specimens.^7^, ^8^, ^9^

The majority (>50%) of lung cancer cases do not have a surgical biopsy or resection specimen for ancillary testing and instead rely primarily on fine needle aspiration biopsy (FNAB) specimens for diagnosis, staging and ancillary tests.^10^ Not surprisingly, a review of the literature shows multiple studies exploring the feasibility of PD‐L1 immunostaining on cytological samples. In addition, there are numerous cytology studies that address pre‐analytical (e.g., different fixatives), analytical (e.g., antibody clone, staining platforms, inter and intra‐observer agreement, cytology‐histology concordance) and post‐analytical (e.g., clinical outcome) issues.^11^, ^12^, ^13^, ^14^, ^15^, ^16^ Although promising results have emerged, many important questions about this field still need to be addressed.

This review summarizes the literature of PD‐L1 immunostaining in lung cytological specimens and provides practical tips for optimizing PD‐L1 analysis.

## 2 METHODS

A systematic data search was undertaken using PubMed and Embase electronic databases from January 2017 until April 2021. Key words including “cytology” and “PD‐L1” were used for this data search. We were interested in the current status of PD‐L1 testing in lung cytology specimens with respect to pre‐analytic, analytic and post‐analytic aspects of this test. Exclusion criteria included animal studies, PD‐L1 testing other than IHC/ICC such as flow cytometry or molecular methods, studies not dealing with lung carcinoma, or studies without any comparisons like cytology‐histology concordance, inter‐observer comparison or clone comparison. No language restrictions were applied. Different study designs, different antibody clones and staining platforms employed, as well as variations in study population precluded a formal meta‐analysis of these data.

## 3 RESULTS

After deleting duplicates, 4730 articles were screened. A total of 77 full‐text and 30 abstract articles matched study goals. Studies analyzing PD‐L1 testing on lung carcinoma cytology specimens originated mostly from the USA, followed by publications arising from Italy, Canada, United Kingdom, China, Germany, Japan, India, Turkey and other countries. The Dako clone 22C3 was the most common clone analyzed on cytology specimens, followed by the Ventana SP263 clone.^17^, ^18^, ^19^, ^20^, ^21^, ^22^, ^23^, ^24^, ^25^, ^26^, ^27^, ^28^, ^29^, ^30^, ^31^, ^32^, ^33^, ^34^, ^35^, ^36^, ^37^, ^38^, ^39^, ^40^, ^41^, ^42^, ^43^, ^44^, ^45^, ^46^, ^47^, ^48^, ^49^, ^50^, ^51^, ^52^, ^53^, ^54^, ^55^, ^56^, ^57^, ^58^, ^59^, ^60^, ^61^, ^62^, ^63^, ^64^, ^65^, ^66^, ^67^ The data collected included the clone type used in each study. Pertinent details covering the technical aspects of the immunostain assay performed were also noted. An attempt was made to exclusively include only cases involving FNAB of lung NSCLC with a PD‐L1 test. Many studies included a subset of samples procured from metastatic disease involving the lymph nodes or effusion specimens. As noted, heterogeneity of the various study designs precluded meta‐analysis of the collected data.

## 4 PRE‐ANALYTICAL CONSIDERATIONS

### 4.1 Specimen collection

Newer generation biopsy needles and minimally invasive trans‐bronchial needle aspiration (TBNA) or endoscopic ultrasound‐guided fine needle aspiration biopsy (EBUS‐FNAB) procedures have revolutionized cytology sample procurement. These procedures have become the test of choice for the initial diagnosis, staging and tissue procurement for biomarker testing of lung lesions. Many studies have proven the feasibility of cytology specimens obtained via EBUS or TBNA for PD‐L1 testing.^68^, ^69^, ^70^, ^71^, ^72^, ^73^, ^74^, ^75^, ^76^ Standard needle sizes used for these procedures range from 21 to 25 G.^48^, ^68^, ^69^, ^70^, ^71^, ^72^, ^73^, ^74^, ^75^, ^76^

Sakakibara et al.^66^ compared EBUS‐TBNA from lymph nodes to excised lymph nodes and also to the primary tumors. They demonstrated good concordance with an *R* value of 0.93 for TBNA and lymph node excisions and 0.75 for TBNA versus primary lung tumor resections, for clone AbCam EPR1161. A recent study by Perrotta et al.^22^ studied the effect of assessment of PD‐L1 on TBNA samples that they also compared with other sampling methods such as percutaneous FNA, percutaneous core needle biopsy (CNB), thoracoscopy, excisions by using video‐assisted thoracoscopic surgery (VATS), or open thoracotomy. Needle sizes used in their study were 19G, 21G, 22G and 25G. Sample adequacy between these different methods did not show any statistically significant difference. Davidson et al.^69^ undertook a prospective study using a 19G needle for lymph node aspirates and demonstrated that the majority (14/17) samples were adequate for the evaluation of PD‐L1. In this study, 42.9% of the samples demonstrated positive PD‐L1 expression. Similarly, Wahidi et al.^31^ concluded that the utility of utilizing a 19G needle for PD‐L1 testing and molecular testing without the risk of any increase in adverse events.

There is no published evidence that needle size significantly affects sample adequacy for PD‐L1 testing.^31^, ^62^ Although, a recent study by Hardy et al.^76^ showed that needle size can still affect adequate sample procurement. In this study, PD‐L1 testing failures occurred in 3/5 (60%) 22G needle biopsies, 1/5 (20%) in 21G needle biopsies, and 2/39 (5.1%) in 19G needle biopsies (with a *p* value of .016). These results are skewed due to the number of samples compared (five samples with 21–22 G vs. 39 samples with 19G needle).

### 4.2 Role of rapid onsite evaluation

ROSE by a trained cytotechnologist or cytopathologist increases the success rate of tissue procurement and allows for the appropriate triage of ancillary testing of all cancer types. Indeed, studies by Stevenson et al.^77^ and Doxtader et al.^78^ evaluating ROSE during EBUS procedures confirmed the utility of ROSE to increase the yield of aspirated sample for ancillary tests such as PD‐L1.

### 4.3 Test requisition form

The pathology laboratory should consider providing guidelines for ordering PD‐L1 testing. Test menus accordingly need to ideally incorporate educational material.

### 4.4 Type of cytological samples and fixatives:

The IASLC (International Association for the Study of Lung Cancer)^11^ discusses the use of a variety of cytology sample types for PD‐L1 immunostaining. Of the different cytological preparations available, the cell‐block (CB) is the most common type of processed specimen material extensively studied for PD‐L1 ICC followed by other preparation types such as direct smears (unstained, air‐dried or alcohol fixed), cell‐transfer, cytospins and liquid‐based preparations.^79^, ^80^, ^81^

Of these specimens, CBs are typically handled similar to formalin fixed, paraffin embedded (FFPE) tissues and thus their use is similar to that of FFPE material. However, one of the major pre‐analytical factors that can affect ICC performance of CBs is the variety of methods in which CBs are prepared and fixatives used prior to cell‐blocking that vary for each laboratory. Cell‐block method preparation was not consistently provided for appropriate analysis. Different fixatives used include alcohol, CytoLyt, CytoRich Red, MicroFix spray and formalin, and RPMI or alcohol followed by formalin.^82^, ^83^, ^84^, ^85^ According to some authors, alcohol‐based fixatives might compromise IHC staining.^82^, ^83^, ^84^ However, several studies exploring the effect of different fixatives before cell‐blocking concluded that the type of fixative does not in fact affect PD‐L1 staining. This includes investigations by Wang et al.^35^ about alcohol only, formalin only, and both fixatives, as well as the study by Gosney et al.^32^ about alcohol‐based fixatives like CytoRich Red or CytoLyt and neutral buffered formalin, and the paper by Lou et al.^28^ using CytoLyt. Of the direct smear studies, a study by Lozano et al.^39^ demonstrated good concordance between PD‐L1 expression in FFPE tissue, FFPE CBs and alcohol fixed Papanicolaou stained direct smears. Similar results have been confirmed by other researchers.^46^, ^49^, ^60^ Wang et al.^86^ further showed that destained Papanicolaou smears are less sensitive than CBs in detecting PD‐L1 for clone SP263. Our literature review supports 10% buffered formalin as the fixative of choice for CBs for PD‐L1 ICC. Vigliar et al.^36^ concluded that fixation time affects the performance of a LDT employing the 22C3 PD‐L1 assay, but did not affect the results of a commercially available SP263 assay. Hence, they advocate checking fixation times for LDTs.

### 4.5 Tissue block age

Ideal surgical pathology specimens used for PD‐L1 testing should not be older than 3 years according to a study conducted by Boothman et al.^87^ Studies exploring this aspect on cytology material are lacking.

## 5 ANALYTICAL CONSIDERATIONS

### 5.1 Use of appropriate controls

Use of positive and negative controls remain one of the key elements in the analytical stage for optimal PD‐L1 test performance. Placenta and tonsil were the most common external positive controls used for PD‐L1 testing using cytology material. Some researchers used commercial cell‐lines as positive controls while developing protocols for PD‐L1 expression for cytological samples.^88^

### 5.2 Sample adequacy

Multiple studies have assessed the adequacy rate of PD‐L1 evaluation on cytology samples with reported adequacy rates ranging between 50% and 96%.^89^, ^90^, ^91^, ^92^ Most studies used a cellularity of less than 100 cells as an exclusion criterion for PD‐L1 ICC. A minimum of 100 viable and well‐preserved cells are required to perform PD‐L1 IHC/ICC. Studies have shown that concordance rates vary in direct proportion to the cellularity of the cytology specimens.^61^ In this study, the authors demonstrated that the concordance rate at a cut‐off of 50% was near perfect (>0.80) for cell count of 400 for clone 28–8 and cell count of 500 for clone SP142 compared to moderate concordance at cellularity between 100 and 500 cells.

### 5.3 Currently available PD‐1/PD‐L1 IHC/ICC assays for NSCLCs

Table 1 provides and overview of the different PD‐L1 clones commercially available for IHC and ICC testing. Due to the availability of these different PD‐L1 assays and different staining platforms, coupled with different cut‐off levels to determine the positivity of PD‐L1 expression, standardize PD‐L1 testing and reporting of results remains challenging.

**TABLE 1:** Summary of available commercial monoclonal PD‐L1 antibodies

Assay clone	Staining platform	Target drug name	Drug target	Cell type and location for assessment
22C3	Dako	Pembrolizumab	PD1	Tumor cell membrane
28–8	Dako	Nivolumab	PD1	Tumor cell membrane
SP263	Ventana	Durvalumab	PD‐L1	Tumor cell membrane
SP 142	Ventana	Atezolizumab	PD‐L1	Tumor cell and/or immune cell membrane

Abbreviations: PD, programmed death; PD‐L1, programmed death ligand 1.

### 5.4 PD‐L1 immunocytochemistry interpretation

Table 2 summarizes of interpretation guidelines for cytology material.^11^, ^93^, ^94^, ^95^, ^96^, ^97^

**TABLE 2:** PD‐L1 immunocytochemistry interpretation guideline

*Sample adequacy* ^a^: At least 100 viable, well‐preserved, non‐overlapping tumor cells *Positive stain result*: Complete or partial membranous staining of tumor cells irrespective of the staining intensity *Negative stain result*: Exclusively cytoplasmic staining, granular cytoplasmic staining or nuclear staining of tumor cells *Pitfalls to avoid*: Avoid macrophages and inflammatory cells while scoring tumor cells by parallel examination of H&E stained glass or digital slides *Cytology specimen considerations*: 3‐D clusters (smears), more non‐specific cytoplasmic staining, cellular fragmentation, background cellular debris, inflammatory cell contamination from blood, lack of intact architecture (e.g., cannot count tumor infiltrating lymphocytes) *Consideration of repeat testing*: Too much background staining, weak staining of control *How to improve interpretation*: Consider providing training for PD‐L1 interpretation or participation in proficiency testing
*Sample report format of PD‐L1 on lung cytology specimens*:
The cytology report should include the following information:
*PD‐L1 ICC parameters*:
a. Cytology sample type
b. Clone
c. Staining platform utilized
d. Laboratory developed test (LTD): Yes or No
*PD‐L1 ICC scoring*:
Scoring system used
Exact Score: e.g., Tumor proportion score for Dako 22C3 clone

Abbreviations: ICC, immunocytomistry; PD, programmed death; PD‐L1, programmed death ligand 1.

^a^ A disclaimer should be included if cell‐block cellularity has <100 cells and that repeat sampling may be considered if clinically indicated.

**FIGURE 1:**
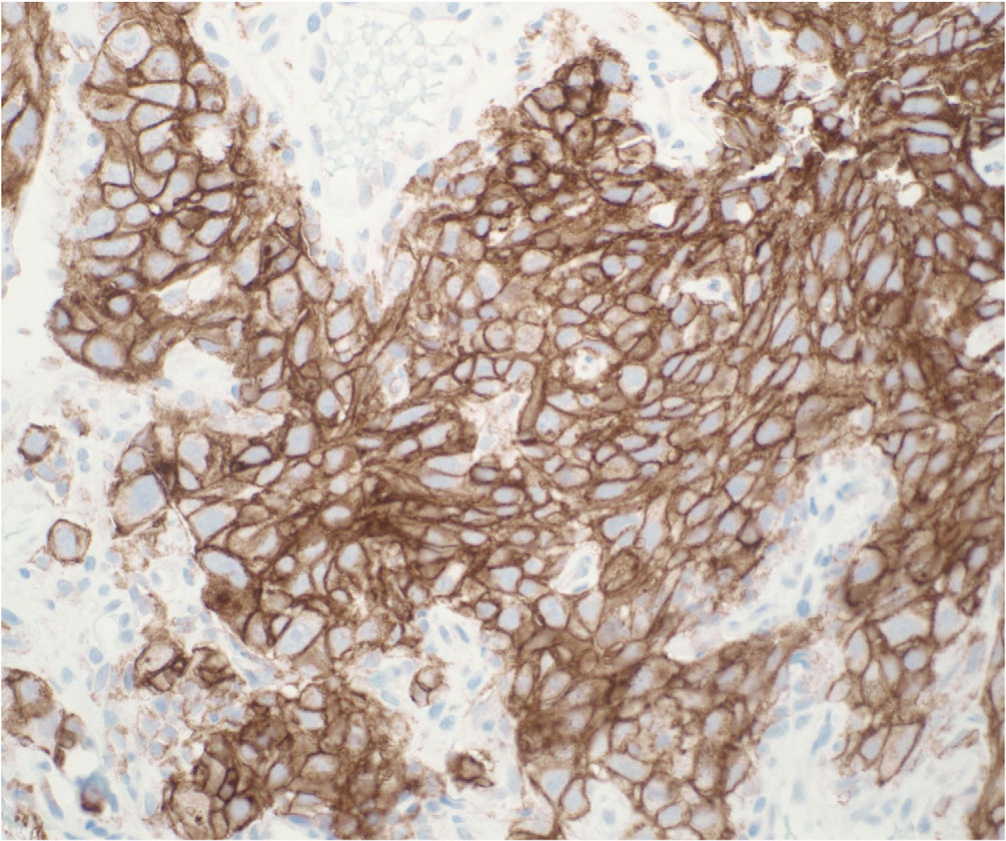
Program death ligand‐1 (PD‐L1) immunostain performed on a cell block section of a non‐small cell lung carcinoma showing diffuse circumferential expression (×200 magnification, clone 22C3, Dako) [Colour figure can be viewed at wileyonlinelibrary.com]

In brief, a literature review indicates that complete or partial membranous staining (Figure 1), irrespective of stain intensity, was considered positive in most studies. Exclusive cytoplasmic staining or granular cytoplasmic staining should be considered as negative. Similarly, nuclear staining should be considered as artifactual and interpreted as negative.

For most assays, only tumor cells are scored whereas for assay SP142 tumor cells and immune cells need to be scored. The SP142 clone is rarely used in cytology due to the inability to establish a true relationship between tumor cells and inflammatory cells in cytology material. The most common scoring system used in the literature includes a 3‐tiered scoring system based on proportion (number of PD‐L1 stained tumor cells divided by the total number of viable tumor cells, multiplied by 100) of tumor cells staining with negative cases (TPS = tumor proportion score < 1), low positive cases (TPS score ≥ 1–49) and high positive cases (TPS score ≥ 50). Caution needs to be taken to exclude inflammatory cells like macrophages while assessing tumor cells for accurate PD‐L1 scoring. Identifying macrophages can be a challenging task for cytology preparations due to the 3‐dimensional nature (applies to aspirate smears only) of cytology samples.^60^ Gagne et al.^38^ specifically analyzed the level of difficulty encountered while scoring PD‐L1 on cytology preparations. They divided the difficulty level into three categories based on the efforts and magnification needed to interpret PD‐L1 staining as positive or negative. They concluded that PD‐L1 scoring on cytology preparations is more cumbersome compared to surgical pathology preparations. Some of the reasons for increased difficulty in scoring PD‐L1 on cytology preparations included the presence of 3‐D clusters leading to overlapping cells on smears that creates difficulty in assessing complete membranous staining, difficulty in separating tumor cells from inflammatory cells (especially pleural effusion specimens), and more cytoplasmic staining or background staining. Similarly, inflammatory cell contamination from aspirated blood and loss of intact tissue architecture remains limiting factors for PD‐L1 evaluation in cytology samples.^13^ Factors to consider for improving the interpretation of PD‐L1 in cytology material include comparing cellular material to the original H&E stained slide of the cell‐block or scanned digital images in parallel during evaluation. Gilani et al.^98^ demonstrated that double staining (TTF1 and PD‐L1; p40 and PD‐L1) was very helpful, easy to perform, and more efficient in evaluating PD‐L1 in cytologic preparations. An important limitation to keep in mind while using double staining with TTF‐1 and PD‐L1 is that >30% of lung adenocarcinomas can be negative for TTF‐1. Although many authors^64^ consider using similar cut‐offs for starting anti‐PD therapy in cytology material to those employed in surgical/excision specimens, this issue warrants further clinical trials exploring clinical outcomes based on PD‐L1 testing primarily performed on cytology specimens using similar versus modified cut‐offs to surgical specimens.

### 5.5 Validation and optimization of laboratory developed tests

The CAP provides guidelines for validation of immunohistochemical stains.^99^, ^100^ Appropriate validation of LTDs and optimization of protocols should be undertaken for achieving diagnostic accuracy comparable to the reference gold standard. The CAP recommends the use of at least 10 positive and 10 negative cases for initial validation and achieving a concordance rate of >90% between the new IHC and comparator IHC. Specific CAP guidelines specific to PD‐L1 IHC are in progress. Illie et al.^41^ developed a 22C3 Antibody concentrate‐based LDT that showed a high concordance rate between tissue biopsy (approximately 100%) and cytology (nearly 95%) specimens when compared to PD‐L1 IHC expression determined using the PD‐L1 IHC 22C3 companion assay at both TPS cut points (≥1%, ≥50%). Skov et al.^21^ explored the effect of different staining platforms (Autostainer and Omnis) for clone Dako 22C3. This demonstrated concordance of 0.99 between the different staining platforms used for clone 22C3.

### 5.6 Cytology‐histology correlation

Many investigators study design included matched or un‐matched cytology‐histology concordance in addition to other end points to explore the feasibility of minimally invasive, rapid cytology samples for PD‐L1 testing compared to recommended more invasive biopsy or resection specimens.^23^, ^101^, ^102^, ^103^, ^104^, ^105^, ^106^, ^107^, ^108^, ^109^, ^110^, ^111^ Concordance rates and k‐values were variably provided, including separate concordance for different cut‐offs and/or overall concordance. Sample size varied from 21 to 247 cases, and the concordance rates varied from 53^17^, ^23^ to almost 100%^46^ (Table 3). Some of the reasons for variable concordance reported in the literature include intra‐tumoral heterogeneity of PD‐L1 expression, variable cellularity, and more 3‐dimensional cell clusters in cytology samples. A study conducted by Shen and Li^101^ analyzed associations between different specimen types and histopathological characteristics and demonstrated significant heterogeneity not in different tumor subtypes, but between primary and metastatic sites and different sample types attributed to intratumoral heterogeneity. A review article by Gosney et al.^12^ evaluated this concordance rate using nine studies, with a total 428 specimens, to demonstrate concordance rates of 88.3% and 89.7% for a TPS cut off of >1% and ≥50%. Overall, these studies confirm the feasibility of cytology specimens for reliable PD‐L1 evaluation. Other rare clones studied by Sakakibara and Russel‐Goldman showed similar results.^66^, ^67^

**TABLE 3:** Summary of published studies assessing cytology‐histology concordance for PD‐L1 testing of non‐small cell lung carcinoma patients

Reference	Number of specimens	Antibody clone	Cytology‐histology concordance rate (kappa)
Ambrosini et al.^17^	26	22C3	53.8% (*k* = 0.31)
Koomen et al.^18^	47	22C3, SP263	57% (*k* = 0.49; 67% (k 0.590
Kuempers et al.^23^	247	22C3	74.1%
Jug et al.^25^	53	22C3	81.5% for adenocarcinoma, 76% for squamous cell carcinoma (*k* = 0.45)
Lou et al.^28^	81	22C3	63% (*k* = 0.68)
Bortolloto et al.^29^	20	22C3	90%
Wang et al.^35^	34	22C3	91.2% (34 samples from different sites) and 100% (16 samples from same anatomic site)
Tsunoda et al.^37^	30	22C3	86.7%
Lozano et al.^39^	113	22C3	97.3%
Ilie et al.^41^	70	22C3	97%
Xu et al.^42^	52	22C3	*k* = 0.54 (adenocarcinoma), *k* = 0.34 (squamous cell carcinoma)
Wang et al.^43^	29	22C3	Pearson correlation 0.925
Noll et al.^46^	28 smears and nine cell block	22C3	High
Arriola et al.^47^	30	22C3	80% (smears), 94.4% (cell blocks) and 62% (cell transfer from Pap stained smears)
Biswas et al.^48^	50	22C3 28–8 SP263	*k* = 0.554 *k* = 0.698 *k* = 0.908
Capizzi et al.^49^	21	22C3	93%
Chauhan et al.^51^	40	SP263	82%
Gagne et al.^52^	46	SP263	*k* = 0.56–0.82
Bozzetti et al.^53^	52	SP263	92.3% (*k* = 0.731)
Ricci et al.^54^	150	SP263	*k* = 0.534 (cut off of 1%) and *k* = 0.740 (>50% cut off)
Pak, Roh	58	SP263	94.34
Daverio et al.^56^	138	SP263	*k* = 0.564
Hendry et al.^57^	58	SP263	*k* = 0.39
Munari et al.^59^	55	SP263	90.6 (50% cut off), 81.1% (1% cut off)
Jain et al.^60^	26	SP263	88.4%
Dong et al.^61^	112	28–8	*k* = 0.377–0.686
Skov^64^	86	28–8 22C3	90% (50% cut off), 87% (1% cut off); 94% (50% cut off), 85% (1% cut off)

Abbreviation: PD‐L1, programmed death ligand 1.

### 5.7 Intra‐ and inter‐observer reproducibility

Very few studies exclusively analyzed intra‐ and/or inter‐observer reproducibility on paired cytology‐histology specimens and reported good reproducibility (Table 4). Of these studies, Munari et al.^59^ reported good inter‐observer concordance (90.5%) and excellent intra‐observer concordance (98.1%) for the SP263 clone. However, Keumpers et al.^23^ reported higher inter‐observer variability in cytology samples compared to histology. Few studies reported better concordance with a cut‐off of TPS ≥50% compared to a cut‐off of 1%. Certain studies explored the impact of different assay types.^18^ Reasons for the variable interpretation are discussed in detail in the analytic considerations, part 4 interpretation of PDL1 ICC of this manuscript.

**TABLE 4:** Summary of published studies assessing inter‐observer agreement/concordance for PD‐L1 testing of non‐small cell lung carcinoma patients

Reference	Preparation type	Number of specimens	Number of pathologists	Antibody clone	Interobserver concordance
Koomen et al.^18^	Cell block	47	2	22C3	High
Sinclair et al.^19^	Cell block	86	5	22C3	Fleiss' kappa (0.74–0.79) and Cohen's kappa (0.49–0.83 to 0.63–0.90)
Hernandez et al.^26^	Cell block	54	3 (cytopathologists with added pulmonary pathology expertise) 4 (without pulmonary expertise)	22C3	42.8% and 61.9% concordance for 21 samples by 7 observers and 3 observers with added pulmonary expertise
Veroceq et al.^27^	Cell block	NA	2	22C3	Discordance rate 16–17.5%
Lou et al.^28^	Cell block	81	2	22C3	0.93–0.97
Krovstov O et al.^30^	Cell block	50	3	22C3	Fliess's kappa 0.66
Heyman et al.^50^	Cell block		Not provided	22C3	93%
Gagne et al.^52^	Cell block	46	4	SP263, 28–8	Fliess's kappa 0.74 to 0.82
Daverio et al.^56^	Cell block	40	2	SP263	0.450
Munari et al.^59^	Cell block	47	2	SP263	90.5% concordance, *k* 0.69
Tsao et al.^102^	Cell block	22	24	22C3, 28–8, SP142, SP263, 73–10	ICC = 0.78–0.85 Fliess's kappa 0.6–0.85
Russel‐Goldman et al.^67^	Cell block	56	2	E1L3N	ICC 0.96

Abbreviations: ICC, interclass correlation; PD‐L1, programmed death ligand 1.

### 5.8 Concordance among assays

Studies exploring the effect of different clones affecting PD‐L1 interpretation in cytology material are limited. Sapalidis et al.,^34^ Lozano et al.^39^ and Ilie et al.^41^ have all reported excellent concordance rates of 99 (Dako 22C3 and Biocare CAL10), 92.7 (22C3 and SP263) and 99 (22C3 antibody concentrate and Dako 22C3 clone), respectively. Gagne et al.^52^ showed a k value of 0.59 (1% cut off) and 0.73 (50% cut off) for clones Ventana SP263 and Dako 28–8. Similar results were shown by Skov et al.^21^ for clones Dako 22C3 and 28–8 with a correlation of 0.95. These findings are further supported by a Blueprint PD‐L1 IHC Comparability Project^102^ in which 24 pulmonary pathologists from 15 countries scored 22 cytology specimens that showed an ICC of 0.78 (cytology glass slides) and 0.85 (cytology digital slides) for different IHC assays and an ICC of 0.89 (surgical glass slides) and 0.93 (surgical digital slides). More dedicated studies using cytology material are needed to address PD‐L1 assay interchangeability for clinical purposes.

### 5.9 Clinical correlates

Some studies compared PD‐L1 expression to clinical characteristics such as age, gender, smoking history, specimen anatomic site (primary versus metastatic), type of NSCLC, cancer stage at diagnosis, and molecular findings. They demonstrated variable associations in the different studies. A complete discussion is beyond the scope of this article. However, Wang et al.^112^ study showed that NSCLC in higher stages is more likely to express PD‐L1, especially in metastases, for clone 22C3. Sakata et al.^44^ compared the effect of neoadjuvant therapy versus no neoadjuvant therapy effect on PD‐L1 and demonstrated a concordance rate of 84% between those groups at a cut off of >50%.

### 5.10 Role of digital pathology

In recent years, we have witnessed the rise of digital pathology and an increasing adoption of this technology in medical centers. This has given researchers and industry the opportunity to explore different image analysis algorithms to address several issues related to manual quantitative PD‐L1 scoring.^19^, ^113^ However, only a few computer applications have been considered for PD‐L1 scoring in cytology until now, and we expect an increase in development of digital automated scoring in the near future, which may help standardize the interpretation of PDL1 in cytology.

## 6 POST‐ANALYTIC CONSIDERATIONS

### 6.1 Correlation with clinical outcomes

There are limited studies^114^, ^115^, ^116^, ^117^ analyzing the actual impact of PD‐L1 analysis on cytology specimens with specific cut‐offs with any particular immunotherapy drug and correlation with clinical outcomes. Studies by Torous et al.^45^ and Stanowska et al.^117^ compared clinical outcome based on cytology specimens and compared their findings to surgical specimens using similar cut offs. They found no significant difference in disease control rates between cytology and surgical specimens. Lozano et al.^116^ also showed a variable response rate to immunotherapy based on cytology samples. Kovacevic et al.^115^ in their study concluded that cytological samples from metastases have the poorest predictive value for assessment of immunotherapy treated patients. Additional studies exploring the effect of using cytology as surrogate material for clinical decision making to start anti‐PD‐L1 therapy are clearly needed.

### 6.2 External quality assurance for inter‐laboratory test performance

Validation of LDTs remain challenging for variety of PD‐L1 assays. External quality assurance for inter‐laboratory test performance may be considered if feasible.^118^, ^119^

## 7 CONCLUSION

Our literature review demonstrates the feasibility of PD‐L1 testing utilizing lung cancer cytology specimens. Published studies report a moderate degree of cytology‐histology concordance, as well as good concordance among different assays. This may likely be attributed to the fact most evaluations of PD‐L1 used cell block preparations. Nevertheless, cytological fixatives do not appear to compromise PD‐L1 staining which further supports the utility of employing minimally invasive, rapid, cost‐effective cytology specimen procurement for biomarker assessment. Reasons that account for variable concordance between cytology and surgical specimens include intra‐tumoral heterogeneity of PD‐L1 expression, cellularity, and more 3‐dimensional cell clusters in cytology samples. Unfortunately, different study designs precluded a robust comparison of results to perform a formal meta‐analysis and thereby draw stronger conclusions. One of the limitations of this review article is that some of the articles were only abstracts that were non‐peer reviewed. Further work still needs to be done to establish standard guidelines for PD‐L1 testing of cytology specimens that address pre‐analytical, analytical, and post‐analytical parameters.

## CONFLICT OF INTEREST

None.

## AUTHOR CONTRIBUTIONS

*Concept and design*: Liron Pantanowitz and Albino Eccher. *Data collection and analysis*: Swati Satturwar and Ilaria Girolami under supervision of Liron Pantanowitz. *Writing first draft*: Swati Satturwar. *Manuscript review and editing*: Ilaria Girolami, Enrico Munari, Francesco Ciompi, Albino Echher and Liron Pantanowitz.

## PRACTITIONER POINTS

This review demonstrates the feasibility of PD‐L1 testing utilizing lung cancer cytology specimens with a moderate degree of cytology‐histology concordance and good concordance among different assays. Cytological fixatives do not appear to compromise PD‐L1 staining which further supports the utility of employing minimally invasive, rapid, cost‐effective cytology specimen procurement for biomarker assessment.

## Data Availability

Data will be available upon request to the corresponding author.
